# Rare polyandry and common monogamy in the firefly squid, *Watasenia scintillans*

**DOI:** 10.1038/s41598-020-68006-1

**Published:** 2020-07-03

**Authors:** Noriyosi Sato, Sei-Ichiro Tsuda, Md. Nur E. Alam, Tomohiro Sasanami, Yoko Iwata, Satoshi Kusama, Osamu Inamura, Masa-aki Yoshida, Noritaka Hirohashi

**Affiliations:** 1 Oki Marine Biological Station, Shimane University, 194 Kamo, Okinoshima, Oki, Shimane 685-0024 Japan; 2 Department of Fisheries, School of Marine Science and Technology, Tokai University, Shizuoka, 424-8610 Japan; 3 Department of Applied Life Sciences, Faculty of Agriculture, Shizuoka University, 836 Ohya, Shizuoka, Shizuoka 422-8529 Japan; 4 Atmosphere and Ocean Research Institute, The University of Tokyo, 5-1-5 Kashiwanoha, Kashiwa, Chiba 277-8564 Japan; 5 Uozu Aquarium, 1390 Sanga, Uozu, Toyama 937-0857 Japan

**Keywords:** Behavioural ecology, Sexual selection

## Abstract

In cephalopods, all species are considered to be polyandrous because of their common life history and reproductive traits reflecting a polyandrous mating system. Contrary to this belief, here we show several lines of evidence for monogamy in the firefly squid, *Watasenia scintillans*. In this species, females are capable of long-term storage of spermatangia, and of egg spawning even after the complete disappearance of males following the breeding season. The stored spermatangia are distributed equally between bilateral pouches under the female’s neck collar. Such a nonrandom pattern of sperm storage prompted us to hypothesize that females might engage in lifetime monandry. Hence, we genotyped female-stored spermatangia and offspring, and found that in 95% of females (18/19), all the spermatangia had been delivered from a single male and all the embryos in a clutch had been sired by spermatozoa from stored spermatangia. In males, throughout the reproductive season, relative testis mass was much smaller in *W. scintillans* than in all other cephalopods examined previously. The mean number of male-stored spermatophores was ~ 30, equivalent to only 2.5 matings. Our genetic, demographic and morphometrical data agree with a mathematical model predicting that monogyny is favored when potential mates are scarce. Together, these results suggest mutual monogamy in *W. scintillans*.

## Introduction

In recent decades, research on behavioral and molecular ecology has identified polyandry (a single female mating with multiple males) as a prerequisite for postcopulatory sexual selection, and as a prevalent mating pattern across animal taxa^1–5^. In this pattern, females are able to receive direct or indirect benefits to a greater extent by mating with multiple males than with a single one, resulting in offspring with “good genes” or higher genetic diversity^6,7^. It is also known that the costs of polyandry (or polygamy) are borne by both sexes through increased predation risks, disease/virus infection, and harassment during courtship or copulation, which often shortens their lifespan^8–10^. Conversely, monogamy (each individual mating with only one partner) as a whole, should meet the criteria of “mutual benefits” for both sexes^7,11,12^ or “unilateral benefit” to one sex over the other (e.g., postcopulatory mate guarding). The latter behavior could also generate sexual conflict or energetic costs to the guarding individuals^13,14^. Hence, monogamy is favored only in environmental conditions where the opportunity or benefit to monopolize mates does not exist^15^. For example, birds and mammals have adopted biparental care for young and long-term (prolonged) pairing, which sets a precondition for the evolution of monogamy^14,16,17^. In addition, species with habitat constraints on feeding or breeding often choose monogamous relationships to protect these resources^18,19^. Furthermore, female dispersal could be a reason for monogamy because searching for extra-pair mates is costly and risky for males^20^. However, monogamy could have arisen in species without these conditions, and still poses an evolutionary puzzle^21^.

Most coleoid cephalopods have a short lifespan (typically range from 6 months to 2 years), reproduce semelparously and display a diverse array of mating behaviors in favor of adaptive or alternative consequences of intrasexual reproductive competition^22–25^. Behavioral and anatomical observations have revealed that some coastal species exhibit coherent reproductive tactics and associated traits in the context of promiscuous mating^22,26–30^. Notably, all cephalopod species reported hitherto are considered to be polyandrous^31–34^ possibly because of the absence of environmental constraints for monogamy. One exception to this is the diamond squid, *Thysanoteuthis rhombus*, which appears to form long-term pair bonds during migration and is possibly monogamous^35^. Although this phenomenon needs to be validated by genetic analyses, many other species also require further investigations to determine whether female promiscuity impacts not only their mating behavior, but also the paternity of their progeny. It is widely recognized that apparent mating behavior—regardless of whether it is promiscuous or monogamous—does not always equate to genetic parentage^3,36^. Nevertheless, it is generally thought that most cephalopods have developed life histories and reproductive modes suitable for pursuing polyandry: thus, they are short lived, semelparous and capable of long-term storage of multiple sperm packages in the female body^34^. In addition, to our knowledge, there have been no reports of biparental care or provisioning by cephalopod males after mating. Postcopulatory mate guarding is a possible cause of mutual monogamy, if it can prevent the opportunities for seeking additional mates by both sexes. Mate guarding by males is often seen in squid and cuttlefish species^37–40^. However, it is either temporal or incomplete, and is sometimes interrupted by extra-pair copulations^39^. Meso- and bathypelagic cephalopods living in the aphotic zones might suffer from low mate availability—another possible cause of monogamy—because of low population density^41–43^. However, the mating patterns of these species are yet to be determined. Although there are many untested scenarios or conditions where monogamy is selected preferentially, in the current consensus, monogamy is regarded as unlikely to be a prevalent strategy in cephalopods^32,33^.

However, most knowledge of reproductive ecology in cephalopods has been obtained from studies with limited representatives—mainly those with coastal habitats—because behavioral observations are possible in field or in aquaria, or limited observations, such as ROV observations^44^ or the examination of cephalopod postmating signals of collected specimens^42,43^. Besides these approaches, DNA fingerprinting is a promising technique to track the outcomes of mating events. To gain global insights into cephalopod reproductive systems, in this study we chose the firefly squid, *Watasenia scintillans* because its ecological characteristics are clear^45^, yet its mating mode remains equivocal. Furthermore, these squid are commercially important resources, which allows fisheries to catch large numbers on a daily basis during the reproductive season^46^. Traditionally in Toyama Bay, the “firefly squid fishery” embargo is lifted on March 1st, which is closely related to unique spawning behaviors of this species. The firefly squid is distributed in the western North Pacific, mainly in the Sea of Japan, Sea of Okhotsk and along the Pacific coast of Japan^45^. Adults occur near the seafloor at depths of > 200 m during the day and migrate upward to depths of 50–100 m at night^45^. Females store sperm within male-derived spermatangia that are affixed to the seminal receptacle located under the collar on the bilateral sides of the nuchal cartilage, and spawn several thousands of eggs at a night and perhaps several times during the spawning season^47^, whereas males do not appear, therefore do not engage in this spawning activity. Here, we show genetic, morphometrical and demographic evidence for a substantial trend in monogamous mating in *W. scintillans*, providing the first reported case in cephalopods.

## Results

### Seasonal dynamics of population and reproduction in *W. scintillans*

**Figure 1:**
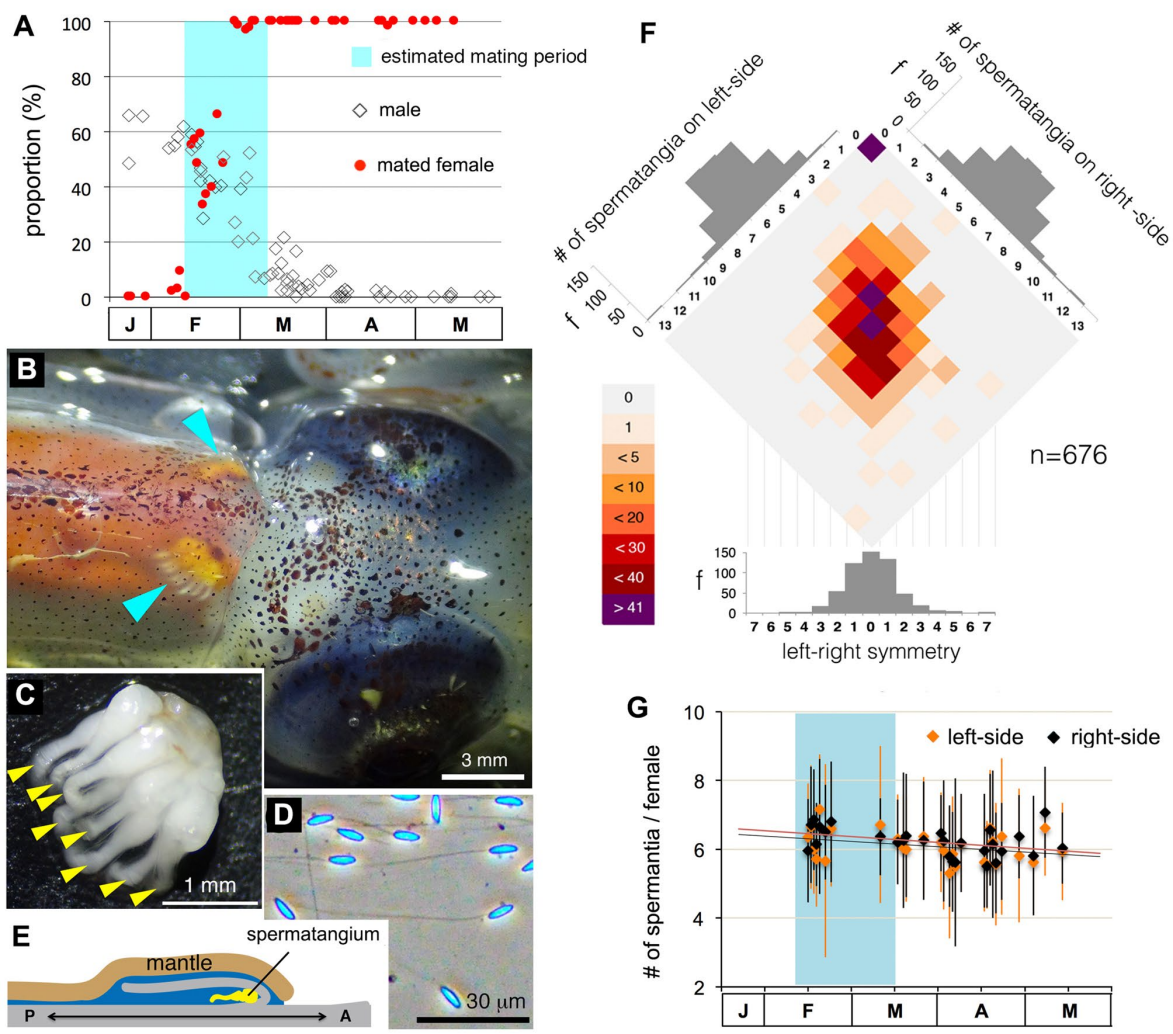
The mode of and change in sperm storage throughout the reproductive season in *W. scintillans.* (**A**) Population dynamics of males and mated females from January to May. A *cyan* box indicates the estimated mating period. Total of 1,414 males and 4,733 females were analyzed. (**B**) A dorsal view of the female around the neck. *Arrowheads* point to the spermatangia visible through the transparent mantle. (**C**) A single mass of spermatangium with unidirectionally oriented ejaculatory ducts (arrowheads). (**D**) Spermatozoa stored in the spermatangia. (**E**) An anatomical illustration of the female seminal receptacle along the body axis (A, anterior; P, posterior). (**F**) A heatmap represents the frequency of female individuals having different patterns of spermatangium number on bilateral sides of the seminal receptacle. Histograms show frequency distribution of spermatangium number for each side (*upper left* and *upper right*) and left–right symmetry (*bottom*). (**G**) No seasonal change in number of spermatangia stored in females. Total of 1,400 females were examined.

We examined seasonal changes in sex ratios and the incidence of female virginity (i.e., absence of any stored spermatangia) in the *W. scintillans* population caught by regional trawl-fishing around the Oki Islands, in the Sea of Japan. We found rapid disappearance of males and virgin females in the spring period between mid-February and mid-March (hereafter designated as the estimated mating period, EMP). Noteworthy, in mated females, equivalent numbers of spermatangia, approximately six (left side, 5.89 ± 1.58, max = 13; right side, 5.96 ± 1.65, max = 12; paired Student’s t test, t = –1.02, df = 582, *p* = 0.31), were stored on each side of the seminal receptacle (Fig. 1B–F). This pattern remained unaltered with a constant and gradual decrease in the number of spermatangia throughout the reproductive season (the number of days required to lose one spermatangium from either the left or the right side estimated from a regression line was 175.4 or 192.3, respectively; Fig. 1G), suggesting a lifelong preservation of the spermatangia once attached to the female’s seminal receptacle.

### Evidence for behavioral and genetic monogamy

**Figure 2:**
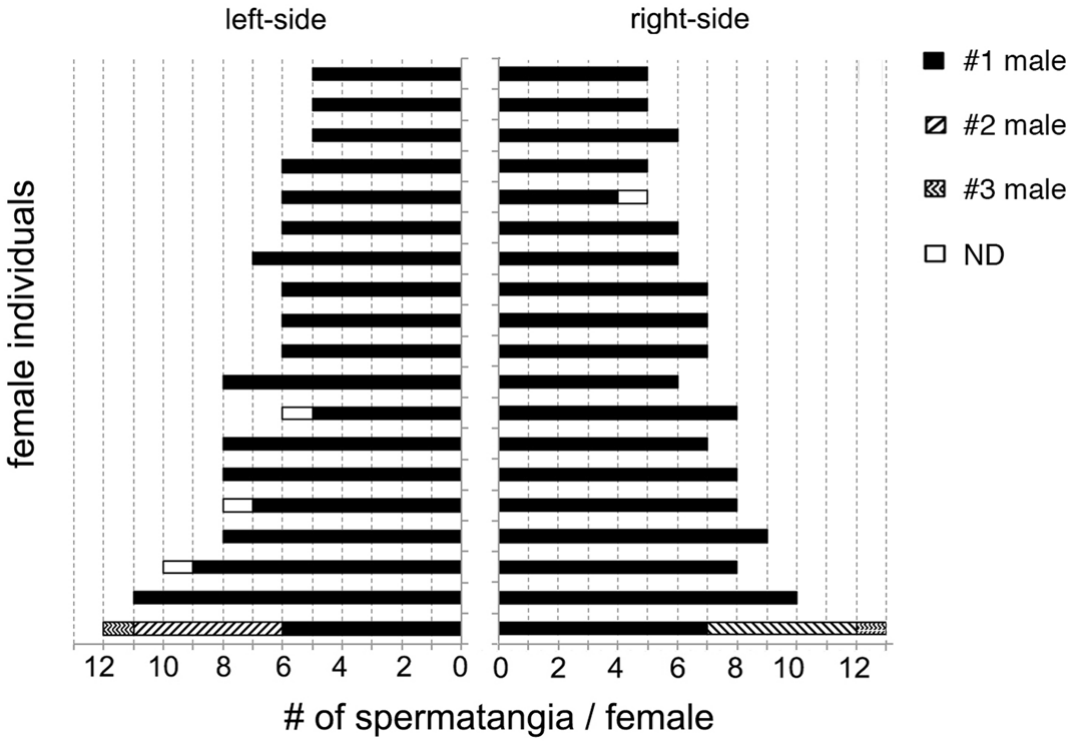
Paternity analysis of each spermatangium stored on the females. Using a total of nineteen females, every single spermatangium was isolated and genotyped. Each column represents paternity share by the first (*filled box*), second (*diagonal stripes*) and third (*jagged stripes*) male. *Open box* indicates unidentified paternity.

The well-organized pattern of sperm storage with left–right symmetry and an approximately fixed number of spermatangia led us to test the hypothesis that females receive spermatozoa by a single copulation from a single male squid (i.e., behavioral monogamy). Through genotyping with validated microsatellite markers (Supplementary Table S2), we found that in 18/19 females (95%) all the stored spermatangia originated from a single male. In one outlying case where the number of spermatangia stored was extraordinarily large (13 on the left and 12 on the right), three males had been copulated, perhaps at different time points (Fig. 2).

To ascertain whether the females used spermatozoa from the stored spermatangia to fertilize their eggs (i.e., genetic monogamy), a DNA-based parentage analysis was carried out using simple sequence repeat (SSR) loci of the mother, her stored spermatangia and her brood. A COLONY analysis^48^ indicated that all paralarvae analyzed had single paternity, derived from the stored spermatangia (Supplementary Datasheet S1, combined nonexclusion probability < 0.001; paternity rate by a candidate male = 100%, n = 4). These results also ruled out the possibility that females might engage in extra-pair copulation and store their sperm in cryptic places other than the seminal receptacle.

### Estimation of male mating opportunities

**Figure 3:**
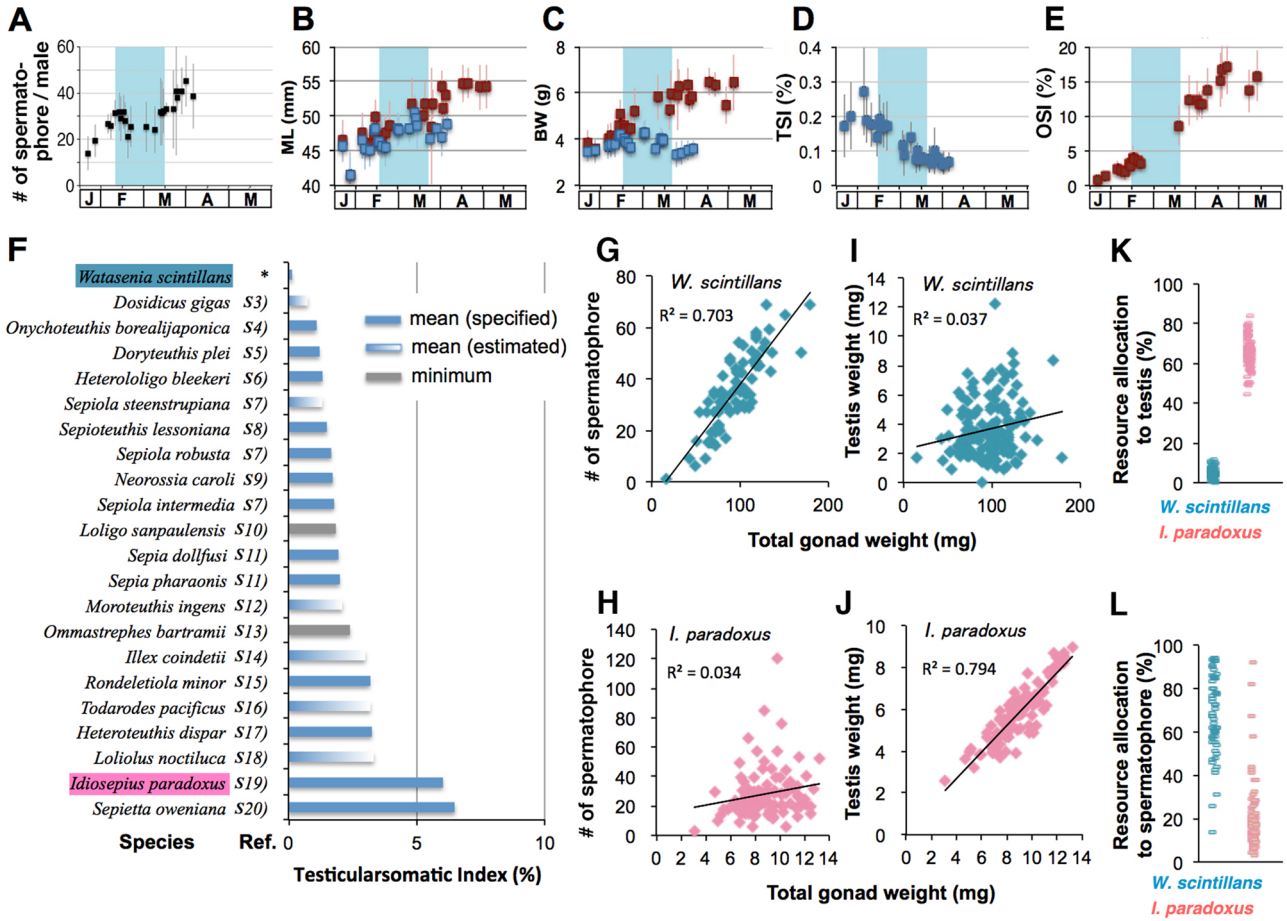
Allocation of male reproductive resources in *W. scintillans* and other squids*.* (**A**–**E**) In *W. scintillans,* seasonal changes in the number of male-storing spermatophore (**A**), mantle length (**B**), body weight (**C**), testicularsomatic indices (**D**) and ovariansomatic indices (**E**) are plotted as the mean ± SE. Data points represent males in *blue* and females in *red*. *Cyan* boxes indicate the estimated mating period. For (**A**–**E**), total of 402 males and 950 females were examined. (**F**) A graph showing a comparison of male testicularsomatic indices among previously reported cephalopod species and *W. scintillans* found in this study (*). Data were extracted from the literature indicated by reference # (Table S1). The columns indicate the mean (*blue column*) or minimum (*gray column*) values specified in the literature, otherwise mean values were estimated (*gradient column*) from presented data points in the graphs. (**G**, **H**) The GSI values from male individuals are plotted against number of spermatophore (**G**, **H**) or testis weight (**I**, **J**) in *W. scintillans* (**G**, **I**) and highly promiscuous *Idiosepius paradoxus* (**H**, **J**). (**K**, **L**) In each individual, allocation of male reproductive resources to testis (**K**) or spermatophore (**L**) is expressed in percentage.

We next performed morphometric and quantitative measurements of the maturity and fecundity of *W. scintillans* individuals before, during and after the EMP. We found that males continue to accumulate spermatophores in their storage organ (Needham’s sac) throughout the season except during the EMP (Fig. 3A) during which males use their spermatophores. Such a continuous increase in the number of stored spermatophores could be seen similarly in the periods before and after the EMP, suggesting that males do not copulate after the EMP. The mean number of male-stored spermatophores just before EMP (pre-EMP) was ~ 30, meaning that males can copulate no more than 2–3 times (because the mean number of spermatophores received by females was 12). At the pre-EMP stage, males become fully mature with the highest testicular–somatic index (TSI), an indicator of sperm-producing capacity or promiscuity (Fig. 3B–D), whereas females have just begun maturing and require several more weeks to become fecund (Fig. 3E). However, to our knowledge, the TSI was much lower in *W. scintillans* (0.15 ± 0.09, n = 408) than in any other cephalopod species reported previously (Fig. 3F). From a different perspective, *W. scintillans* males appear to invest most gonadal expenditure in the production of spermatangia rather than spermatozoa per se, in stark contrast to the case of *Idiosepius paradoxus* (Fig. 3G–L), a species that copulates multiple times throughout the reproductive season^49^. Based on this, we hypothesize that males of *W. scintillans* might have selected a monogynous mating pattern because of their low sperm production capacity and limited mating opportunities caused by a male-biased operational sex ratio and the absence of female remating attempts.

## Discussion

Females of *W. scintillans* come to shallow water to spawn in the spring, while males disappear much earlier from the coastal zones than do females^46^. This means that the lifespan of males is 1 month shorter than in females^45,50^. The coincidental disappearance of males and virgin females supports this scenario as being plausible (Fig. 1A). Hence, females must preserve spermatozoa internally for the considerably long period until spawning.

One of the hallmarks of most coleoid cephalopod reproduction is their copulation behavior, in which males deposit sperm packages (spermatophores) on or inside females using their hectocotylus or terminal organ. Then spermatophores deposited undergo a complex evagination process (spermatophoric reaction) that give rise to stable implantation of ejaculatory apparatus (spermatangium) to female tissue^34,51^. Because mating occurs promiscuously, females receive spermatangia from multiple males simultaneously and/or sequentially, resulting in mixed populations of spermatangia distributed randomly around the deposition sites on the female. However, we consistently found that females of *W. scintillans* store masses of the spermatangia that are evenly distributed at exact locations on bilateral sides of the nuchal cartilage with approximately six on each side. Such an extraordinarily regular pattern of spermatangium placement is unusual in cephalopods^52^. This prompted us to test whether all these spermatangia were derived from a single male.

Our genetic data (the paternity of each spermatangium stored in any one female) clearly demonstrated that most *W. scintillans* females mate with only one male (Fig. 2), suggesting behavioral monandry. However, our results must be interpreted carefully due to occurrence of one exceptional case, where a female mated with three males. Because female promiscuity can be adaptive in response to extrinsic conditions such as an increase of predation risk^53^, it is possible that the firefly squids would also be flexible in choosing mating pattern because of the global range of habitat (from East China Sea to Okhotsk Sea). Furthermore, parentage analysis identified no DNA mismatch between the spermatangia and embryos from the same females, confirming genetic monogamy (Supplementary Fig. S1). This also needs careful interpretation because we analyzed paralarvae with the minimum sample size due to difficulty in culturing embryos until hatching.

During the reproductive season, females can spawn eggs several times at certain intervals^45^. However, other males cannot engage in replenishing the spermatangia during these intervals because: first, males disappear before females are fully fecund (Figs. 1A, 3E); and second, spontaneous loss of once-attached spermatangia to females seldom occurs (Fig. 1G). Accordingly, male mating opportunities are limited by infrequent female remating and male-biased sex ratios. Thus, both tertiary (adult) sex ratios and operational sex ratios^54^ were largely male-biased at the beginning of and throughout the EMP, respectively (Fig. 1A). Under these conditions, mathematical modeling predicts that monogyny (males mating with only one partner) can become fixed as an evolutionary stable strategy (ESS)^54,55^. Therefore, we speculate that female monandry was first established in this species followed by male monogyny, which led to mutual monogamy^54^. If male monogyny becomes an ESS, then evolution could favor males who invest more energy on something other than fecundity (sperm production)^56,57^. This explains the observed low investments in testicular function (sperm production) and spermatangia (copulation opportunity) in this species. Instead of allocating their reproductive investment toward greater fecundity, males could have chosen a “live-fast-die-young” life-history strategy to adapt to a mechanism of first-male sperm precedence^58^ (Fig. 1A). We speculate that this low level of male fecundity arose as a consequence of female-driven monandry^56,57^.

If these female and male squid are predominantly pursuing a monogamous mating strategy, what could be the benefit they reap in exchange for eroding the genetic diversity of their offspring? And in doing so, how do they avoid remating attempts despite being together physically? Therefore, the first question arises as to what might have been a feasible cause (or causes) that drove the mating system in this species to be primarily, if not exclusively, monandrous. It is obvious that some factors known to be involved in monogamy in other taxa do not work for this species, such as long-term pair bonding, biparental care, low population density, habitat limitation, time constraints for reproduction and enforcement (mate guarding)^16,18,21,59^. This is simply because they make condensed spawning migrations and males disappear entirely before reaching the peak of the main spawning season. Alternatively, considering that this species suffers from high predation pressures and serves as the dominant prey for demersal fishes^60^, time-consuming courtship behavior could potentially be deleterious to their survival, so multiple mating would reduce reproductive success for both sexes^8,61,62^. There are ample examples to support the prediction that predation risk serves as an evolutionary force favoring monandry^8^. For example, in the cicada *Subpsaltria yangi*, when males emit an advertising call, only virgin females make a response call, then males fly to them to mate (hence, monandry). These nuptial flights by males have a high risk: only 25% of second flights were successful and the male cicadas who failed were mostly attacked by the robber fly, *Philonicus albiceps*^63^.

It is widespread across taxa that non-virgin (mated) females lose sexual attractiveness or responsiveness to male-specific courtship signaling, and the underlying mechanisms vary along with physiological, physical and behavioral changes upon mating^64–66^. Nevertheless, the fitness consequences for such females appear to be mostly against the risk of predation. Although the actual mating behaviors of deep-sea organisms are largely hidden or unknown, the successful mating of any species should be well adapted to a dark environment^41^. Communication using bioluminescence signaling executed by the Octopodiformes squid, *Taningia danae*, is regarded as potential courtship behavior^67^. *W. scintillans* has special eyes (photoreceptor cells) containing three visual pigments with different maximum wavelengths (~ 471, ~ 484 and ~ 500 nm), possibly allowing them to distinguish conspecific illumination (green) from environmental down-welling light (blue)^68^. It is therefore feasible that bioluminescence in the firefly squid could play a role in courtship signaling or mate search in a once-in-a-lifetime fashion. Here, our fine-scale analysis of seasonal dynamics in demographics, mating status and reproductive indices of each individual from fishery catches demonstrates the reproductive landscape of this species. In summary, for the first time in cephalopods, we provide genetic evidence for a monandrous mating pattern. Its adaptive significance awaits further study in the light of the ecological niche of this species.

## Methods

### Animal and embryo collection

The squid, *W. scintillans* was obtained from fishery catches by bottom trawls towed around the Oki island (Shimane Prefecture) and Sakai-port off (Tottori Prefecture), or by fixed net set around and near the shelf break in the innermost part of the Toyama bay (Toyama Prefecture), Japan. The commercial fishing period of this species is approximately from January to May in Shimane/Tottori, and from March to May in Toyama. All the morphometric measurements were undertaken on specimens collected in Shimane/Tottori during 2016–2020, whereas for microsatellite analysis of the spermatangia and the parentage analysis, live animals caught at Toyama bay were used during March–April of 2017–2019. They were transported under dark conditions to the Uozu Aquarium, where spawning was induced in the aquarium tank at 14 °C in dark or under far-red illumination. Spawning occurred spontaneously in > 50% of individuals when the field-caught females were transported immediately after the catch (Supplementary Fig. S1). Each egg-string retrieved in isolation was then cultured for 4–5 days at 17 °C until hatching and thereafter, paralarvae being picked at random (15–45 specimens/egg-string, n = 4) were genotyped. All procedures performed in the studies were in accordance with the ethical standards of the Animal Research Committee of Shimane University (ARCSU) and all animal experiments were approved by the ARCSU.

### Measurements of growth and reproductive indices

The squid specimens were measured (mostly within one day after fishing) for dorsal mantle length (ML), total wet weight (body weight; BW), testis weight (TW), spermatophoric complex weight (SCW), number of spermatophore stored in the Needham’s sac and the terminal organ, ovary weight (OW) and number of spermatangium on the left and right side of the female seminal receptacle. The number of spermatangium was counted by viewing under a stereomicroscope. Testicularsomatic index (TSI) and ovariansomatic index (OSI) were calculated as TSI = 100  × TW × BW^−1^ and OSI = 100 × OW × BW^−1^, respectively. To score the number of spermatophore, spermatophoric complex was fixed in 10% formalin in seawater, thereafter dissected under a stereoscope. All males of *W. scintillans* were mature or spent (stage V or VI according to classification by^69^). The data for other squid species were extracted from the literature (Supplementary Table S1). Average spermatophore weight (ASW) was estimated from the regression lines of Fig. 2B, C and in each individual, gonadal investment (resource allocation) to spermatophore was calculated as 100 × (SpN × ASW) × (TW + SCW)^−1^, where SpN indicates number of spermatophore stored in the spermatophoric sac. Resource allocation to testis was calculated as 100 ×  TW ×  (TW  + SCW)^−1^. TSI and other morphometric data of *I. paradoxus*^49^, *H. bleekeri*^70^ and *Doryteuthis plei*^71^ were provided from the corresponding authors of previous reports.

### Development of microsatellite markers

Microsatellite makers (GenBank Acc. LC514114 - LC514117) are developed from partial genome sequences of *W. scintillans* obtained by next-generation sequencing (estimated coverage was 10–11 time by whole genome shotgun analysis) shared by National Center for Child Health and Development in Tokyo. The MISA pipeline^72^ was used to identify reads containing microsatellite repeats and primer sets to amplify the repeats. We searched for trimers, tetramers, pentamers and hexamers with sufficient flanking sequences to develop PCR primers. A total of 1632 putative SSRs with repeat size > 60 bp and flanking regions at the both ends were detected and for SSR screening, thereafter we chose 20 primer pairs flanking tetra- and trinucleotide SSR motif with a minimum of 20 repetitions and the expected product size between 140 and 280 bp. Primer3^73^ was implemented to predict primers pairs targeting the flanking regions. PCR amplification was carried out with genomic DNAs isolated from five different individuals and validated by agarose gel electrophoresis. The primer pairs that gave no band or no obvious polymorphism in band size were eliminated. The remains were further analyzed by acrylamide gel electrophoresis with a dozen of DNA samples obtained from different individuals. Consequently, we selected four SSR loci that were fully characterized by fragment length analysis (see below). Nucleotide sequences of microsatellite makers and their characteristics are shown in Supplementary Table S2.

### Paternity analysis

A mass of spermatangia was removed from the female seminal receptacle, soaked in 70% ethanol for 10 min and disassembled into single spermatandium with fine forceps. Each spermatangium was placed in the 1.5 ml test tube, and digested with 50 µl of lysis buffer (10 mM Tris-Cl (pH 8), 500 mM NaCl, 50 mM EDTA, 2% dithiothreitol, 1 mg/ml Proteinase K (Sigma-Aldrich)) at 52 °C for overnight with constant agitation (120 rpm). After centrifugation (14,000 rpm, 10 min, 4 °C), the supernatant was transferred into new test tube followed by phenol/chloroform extraction. Extracted DNA was precipitated by adding 1/10 volume of 3 M sodium acetate (pH 5.2) and × 2.5 volume of 99.5% ethanol, and centrifuged (14,000 rpm, 10 min, 4 °C). After removing the supernatant, the precipitant was washed with 70% ethanol, air-dried, dissolved in 50 µl of water and stored at − 20 °C. Isolation of genomic DNAs from mantle tissue, hatched paralavae were carried out as well. A parentage DNA analysis was carried out with the same protocol by genotyping the mother, her storing spermatangia, and spawned eggs, using the software program COLONY v.2.0.6.5 (see https://www.zsl.org/colony-download-form)^48^.

All genomic DNA samples were run on 1% agarose gel electrophoresis for evaluating quality and quantity. It should be noted that in rare but certain occasion, no DNA was recovered from the spermatangium, which could be explained by the absence of spermatozoa in the spermatangium. This lack of complete recovery of genomic DNA was reflected on the result that the percent of success in PCR amplification with microsatellite markers was 99.0% (1,172/1,184) and overall success of paternity identification was 98.9% (270/273).

Amplification of microsatellite loci was performed with polymerase chain reaction with fluorescently tagged forward primer and non-labeled reverse primer, and approximately 20 ng of genome DNA in 10 µl of reaction mixture (TaKaRa Ex Taq system) with heat denaturing (95 °C, 1 min) followed by 30 cycles of denaturing (95 °C, 30 s), annealing (56 °C, 30 s) and extension (72 °C, 20 s) and terminated by 15 min incubation at 72 °C. Each PCR product was run on 8% mini-slab polyacrylamide gel electrophoresis (Bio-Rad, 130 V constant, 60 min), and gels stained with 0.1 µg/ml ethidium bromide and image processed by ImageQuant LAS 500 (GE Healthcare). After validation of quality, four PCR products (labeled with FAM, Hex, Cy3 and PET) amplified with the same genome DNA were mixed and subjected to fragment length analysis (3130xl Genetic Analyzer, 500LIZ, FASMAC Co. Japan). Then, by using Cervus 3.0^74^, the combined non-exclusion probabilities for all microsatellite loci were calculated to be < 0.0001, which is sufficiently low value for correctly identifying the real sire^75^.

## Supplementary information

Supplementary file1 (DOCX 3587 kb)

## Data Availability

All datasets for information of the SSR markers and the paternity analysis are available in the electronic supplementary material files and newly developed SSR markers have been deposited at the DDBJ/GenBank with Accession No. LC514114 - LC514117.
