# Supplementary material for: Rare polyandry and common monogamy in the firefly squid, Watasenia scintillans
Source: Sci Rep. 2020 Jul 3;10:10962. doi: 10.1038/s41598-020-68006-1 (PMC7334199; doi:10.1038/s41598-020-68006-1)
Supplement: Supplementary file 1 — Supplementary file1 (DOCX 3587 kb) [file 41598_2020_68006_MOESM1_ESM.docx]

**Supplemental data**

**Rare polyandry and common monogamy in the firefly squid, *Watasenia scintillans***

Noriyosi Sato^1,2^, Sei-Ichiro Tsuda^1^, Nur E Alam Md^1^, Tomohiro Sasanami^3^ Yoko Iwata^4^, Satoshi Kusama^5^, Osamu Inamura^5^, Masa-aki Yoshida^1^, Noritaka Hirohashi^1, *^

^1^Oki Marine Biological Station, Shimane University, 194 Kamo, Okinoshima, Oki, Shimane 685-0024, Japan

^2^Department of Fisheries, School of Marine Science and Technology, Tokai University, Shizuoka 424-8610, Japan

^3^Department of Applied Life Sciences, Faculty of Agriculture, Shizuoka University, 836 Ohya, Shizuoka, Shizuoka 422-8529, Japan

^4^Atmosphere and Ocean Research Institute, The University of Tokyo, 5-1-5 Kashiwanoha, Kashiwa, Chiba 277-8564, Japan

^5^Uozu aquarium, 1390 Sanga, Uozu, Toyama 937-0857, Japan

**Supplementary Table S1**

**
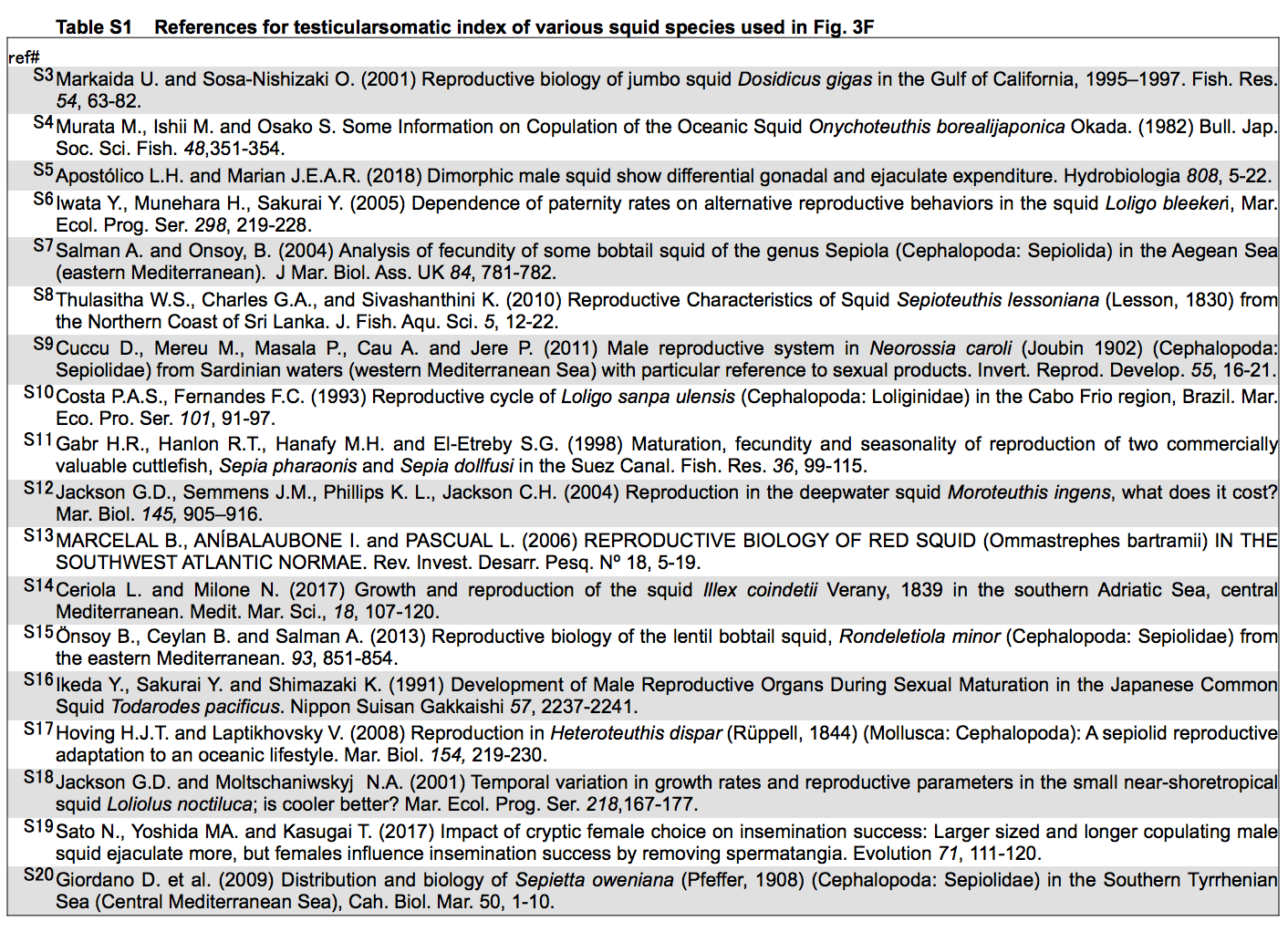
**

**Supplementary Table S2**

**
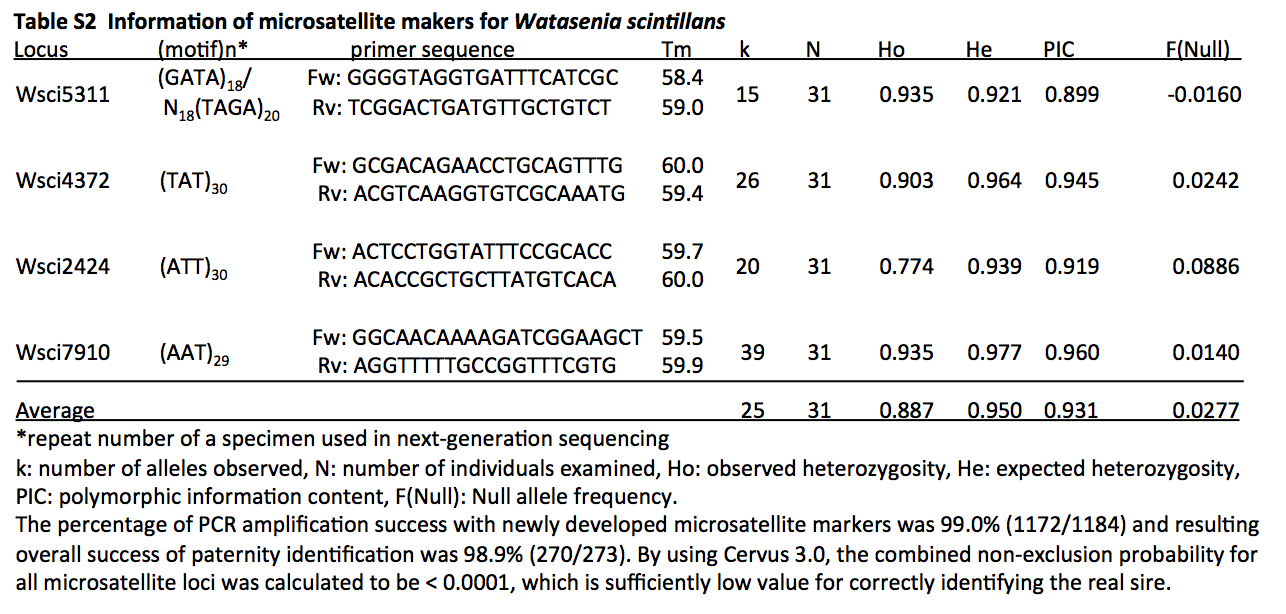
**

**Supplementary Fig. S1**

**
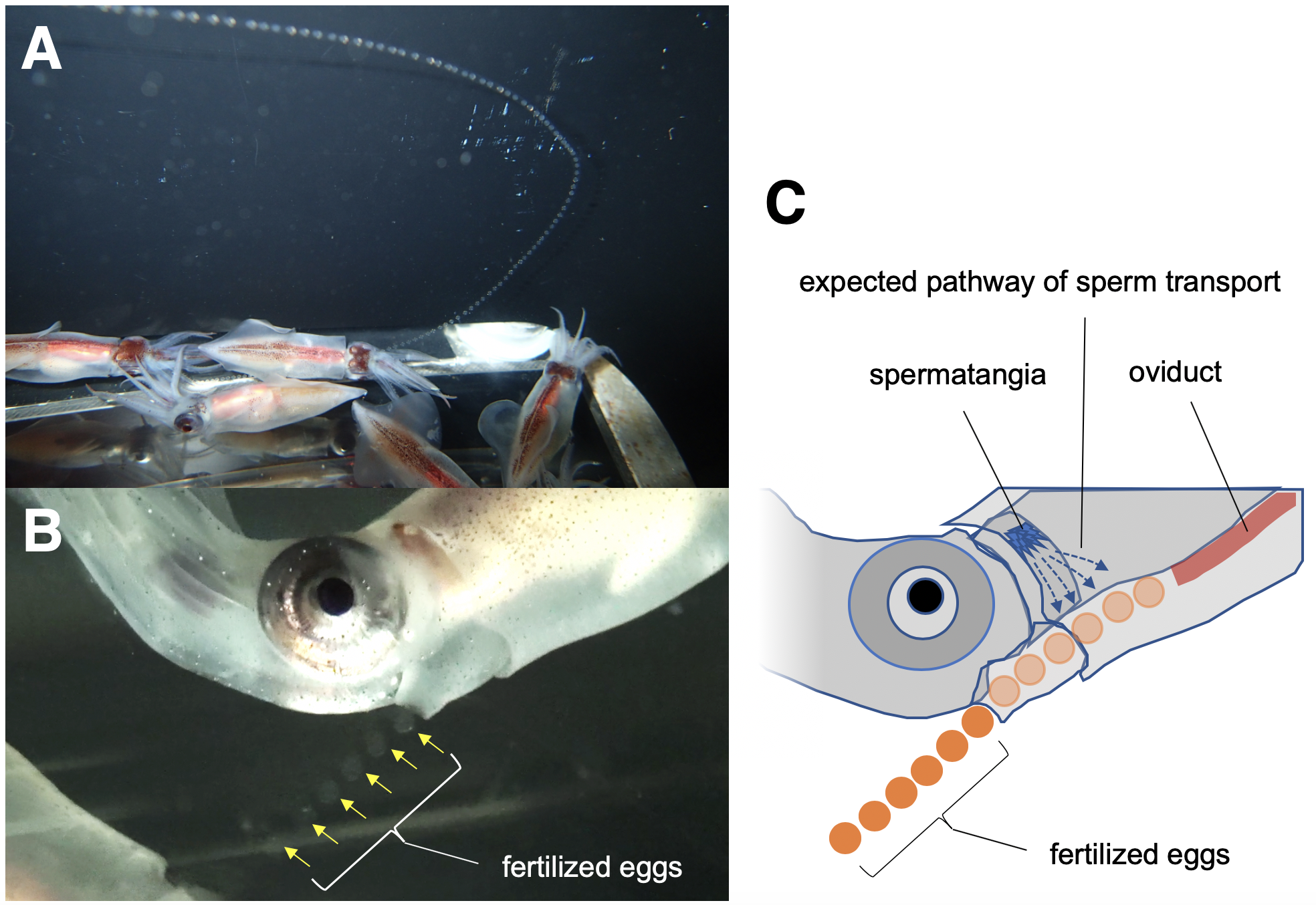
**

**Supplementary Fig. S1**

**Spontaneously induced egg spawning under captive condition.**

A & B, Mature females trigger egg spawning in dark. Upon spawning, hundreds of eggs were released with a filamentous string. C, a diagram shows expected pathway of sperm transport to ovulated eggs. The opening of the oviduct is located adjacent to the ink sac along ventral midline.

**Supplementary Datasheet S1**

| Experiment 1 | Wsci2424 | | Wsci4372 | | Wsci7910 | |  |  |
| --- | --- | --- | --- | --- | --- | --- | --- | --- |
| female mantle | 211 | 215 | 135 | 135 | 204 | 216 |  |  |
| sperm mass | 197 | 208 | 135 | 156 | 114 | 180 |  |  |
| embryo #2 | 197 | 215 | 135 | 156 | 114 | 216 |  |  |
| embryo #3 | 208 | 215 | 135 | 135 | 180 | 216 |  |  |
| embryo #4 | 197 | 215 | 135 | 135 | 114 | 216 |  |  |
| embryo #5 | 208 | 212 | 135 | 135 | 180 | 204 |  |  |
| embryo #6 | 197 | 215 | 135 | 156 | 114 | 216 |  |  |
| embryo #7 | 197 | 215 | 135 | 156 | 114 | 216 |  |  |
| embryo #8 | 197 | 212 | 135 | 135 | 114 | 204 |  |  |
| embryo #9 | 208 | 212 | 135 | 156 | 180 | 204 |  |  |
| embryo #11 | 208 | 212 | 135 | 156 | 180 | 204 |  |  |
| embryo #13 | 197 | 212 | 135 | 156 | 114 | 204 |  |  |
| embryo #15 | 197 | 215 | 135 | 156 | 114 | 216 |  |  |
| embryo #16 | 208 | 212 | 135 | 156 | 180 | 204 |  |  |
| embryo #17 | 208 | 215 | 135 | 135 | 180 | 216 |  |  |
| embryo #18 | 197 | 212 | 135 | 156 | 114 | 204 |  |  |
| embryo #19 | 197 | 215 | 135 | 156 | 114 | 216 |  |  |
|  |  |  |  |  |  |  |  |  |
|  |  |  |  |  |  |  |  |  |
| Experiment 2 | Wsci2424 | | Wsci7910 | |  |  |  |  |
| female mantle | 173 | 185 | 171 | 318 |  |  |  |  |
| sperm mass | 191 | 216 | 105 | 180 |  |  |  |  |
| embryo #1 | 185 | 215 | 105 | 318 |  |  |  |  |
| embryo #2 | 173 | 215 | 105 | 171 |  |  |  |  |
| embryo #3 | 185 | 215 | 105 | 318 |  |  |  |  |
| embryo #4 | 185 | 191 | 180 | 318 |  |  |  |  |
| embryo #5 | 185 | 191 | 180 | 318 |  |  |  |  |
| embryo #7 | 185 | 191 | 180 | 318 |  |  |  |  |
| embryo #8 | 173 | 215 | 105 | 171 |  |  |  |  |
| embryo #9 | 173 | 215 | 105 | 171 |  |  |  |  |
| embryo #10 | 185 | 191 | 180 | 318 |  |  |  |  |
| embryo #11 | 185 | 215 | 105 | 171 |  |  |  |  |
| embryo #12 | 173 | 215 | 105 | 171 |  |  |  |  |
| embryo #13 | 173 | 215 | 105 | 171 |  |  |  |  |
| embryo #14 | 173 | 191 | 171 | 180 |  |  |  |  |
| embryo #15 | 173 | 191 | 171 | 180 |  |  |  |  |
| embryo #16 | 185 | 191 | 180 | 318 |  |  |  |  |
| embryo #17 | 185 | 215 | 180 | 318 |  |  |  |  |
| embryo #18 | 185 | 215 | 105 | 318 |  |  |  |  |
| embryo #19 | 185 | 191 | 180 | 318 |  |  |  |  |
| embryo #20 | 185 | 191 | 180 | 318 |  |  |  |  |
|  |  |  |  |  |  |  |  |  |
|  |  |  |  |  |  |  |  |  |
| Experiment 3 | Wsci2424 | | Wsci5311 | | Wsci4372 | |  |  |
| female mantle | 200 | 204 | 146 | 208 | 116 | 166 |  |  |
| sperm mass | 182 | 200 | 170 | 203 | 107 | 123 |  |  |
| embryo #1 | 182 | 200 | 170 | 208 | 107 | 116 |  |  |
| embryo #3 | 200 | 204 | 170 | 208 | 122 | 166 |  |  |
| embryo #4 | 200 | 200 | 146 | 203 | 107 | 116 |  |  |
| embryo #5 | 200 | 204 | 146 | 170 | 107 | 116 |  |  |
| embryo #7 | 200 | 204 | 170 | 208 | 107 | 116 |  |  |
| embryo #8 | 182 | 204 | 146 | 203 | 122 | 166 |  |  |
| embryo #9 | 182 | 204 | 203 | 207 | 107 | 116 |  |  |
| embryo #10 | 200 | 204 | 170 | 208 | 107 | 116 |  |  |
| embryo #11 | 182 | 200 | 146 | 203 | 107 | 166 |  |  |
| embryo #12 | 200 | 200 | 146 | 170 | 107 | 166 |  |  |
| embryo #13 | 200 | 200 | 203 | 208 | 116 | 122 |  |  |
| embryo #14 | 182 | 200 | 146 | 203 | 107 | 116 |  |  |
| embryo #15 | 182 | 204 | 203 | 208 | 122 | 166 |  |  |
| embryo #16 | 182 | 204 | 170 | 208 | 166 | 122 |  |  |
| embryo #17 | 200 | 204 | 203 | 208 | 116 | 122 |  |  |
| embryo #18 | 200 | 200 | 203 | 208 | 107 | 116 |  |  |
| embryo #19 | 182 | 204 | 146 | 170 | 107 | 166 |  |  |
| embryo #20 | 200 | 204 | 203 | 208 | 116 | 122 |  |  |
|  |  |  |  |  |  |  |  |  |
|  |  |  |  |  |  |  |  |  |
| Experiment 4 | Wsci2424 | | Wsci5311 | | Wsci4372 | | Wsci7910 | |
| female mantle | 150 | 267 | 221 | 225 | 203 | 241 | 266 | 280 |
| sperm mass | 165 | 279 | 204 | 241 | 225 | 251 | 272 | 298 |
| embryo #2 | 150 | 165 | 225 | 242 | 225 | 241 | 280 | 298 |
| embryo #3 | 267 | 279 | 204 | 225 | 203 | 251 | 266 | 272 |
| embryo #4 | 165 | 267 | 221 | 242 | 225 | 241 | 266 | 272 |
| embryo #5 | 150 | 279 | 204 | 225 | 203 | 225 | 272 | 280 |
| embryo #6 | 150 | 279 | 205 | 225 | 203 | 225 | 272 | 280 |
| embryo #7 | 150 | 279 | 205 | 225 | 241 | 250 | 272 | 280 |
| embryo #8 | 150 | 165 | 205 | 221 | 203 | 251 | 280 | 298 |
| embryo #9 | 150 | 165 | 204 | 221 | 203 | 251 | 280 | 298 |
| embryo #10 | 165 | 267 | 221 | 242 | 225 | 241 | 266 | 298 |
| embryo #11 | 150 | 279 | 221 | 241 | 241 | 251 | 272 | 280 |
| embryo #12 | 150 | 279 | 225 | 242 | 203 | 251 | 272 | 280 |
| embryo #13 | 150 | 279 | 225 | 241 | 203 | 225 | 272 | 280 |
| embryo #14 | 267 | 279 | 205 | 225 | 203 | 251 | 272 | 280 |
| embryo #15 | 150 | 279 | 221 | 242 | 241 | 251 | 272 | 280 |
| embryo #16 | 150 | 165 | 204 | 225 | 203 | 225 | 280 | 298 |
| embryo #17 | 165 | 267 | 221 | 242 | 203 | 225 | 266 | 298 |
| embryo #18 | 165 | 267 | 221 | 242 | 225 | 241 | 266 | 298 |
| embryo #19 | 267 | 279 | 221 | 242 | 203 | 225 | 272 | 280 |
| embryo #20 | 150 | 279 | 204 | 225 | 203 | 251 | 272 | 280 |
| embryo #21 | 165 | 267 | 221 | 241 | 225 | 241 | 280 | 298 |
| embryo #22 | 267 | 279 | 221 | 242 | 225 | 241 | 266 | 272 |
| embryo #23 | 150 | 165 | 221 | 242 | 203 | 251 | 280 | 298 |
| embryo #24 | 150 | 279 | 225 | 242 | 241 | 251 | 272 | 280 |
| embryo #25 | 267 | 278 | 205 | 221 | 204 | 251 | 266 | 272 |
| embryo #26 | 165 | 267 | 225 | 242 | 204 | 251 | 266 | 298 |
| embryo #27 | 150 | 279 | 221 | 242 | 225 | 241 | 272 | 280 |
| embryo #28 | 150 | 279 | 225 | 241 | 241 | 251 | 280 | 298 |
| embryo #29 | 165 | 267 | 204 | 221 | 225 | 241 | 266 | 298 |
| embryo #30 | 150 | 165 | 204 | 221 | 241 | 251 | 280 | 298 |
| embryo #31 | 150 | 279 | 204 | 225 | 225 | 241 | 272 | 280 |
| embryo #32 | 267 | 279 | 204 | 221 | 241 | 251 | 266 | 272 |
| embryo #33 | 150 | 165 | 221 | 242 | 241 | 251 | 280 | 298 |
| embryo #34 | 165 | 267 | 204 | 225 | 225 | 241 | 266 | 298 |
| embryo #35 | 165 | 268 | 204 | 225 | 241 | 251 | 266 | 298 |
| embryo #37 | 150 | 279 | 221 | 242 | 241 | 251 | 272 | 280 |
| embryo #38 | 165 | 267 | 221 | 242 | 203 | 251 | 266 | 298 |
| embryo #39 | 165 | 267 | 221 | 242 | 203 | 251 | 280 | 298 |
| embryo #40 | 165 | 267 | 225 | 242 | 203 | 222 | 266 | 298 |
| embryo #41 | 267 | 279 | 204 | 225 | 225 | 241 | 266 | 272 |
| embryo #43 | 150 | 165 | 225 | 242 | 225 | 241 | 280 | 298 |
| embryo #44 | 150 | 279 | 204 | 221 | 241 | 251 | 272 | 280 |
| embryo #45 | 267 | 279 | 204 | 221 | 203 | 225 | 266 | 272 |
| embryo #46 | 150 | 165 | 225 | 241 | 225 | 241 | 280 | 298 |
| embryo #47 | 267 | 279 | 204 | 221 | 203 | 225 | 266 | 298 |
| embryo #48 | 150 | 279 | 204 | 221 | 203 | 225 | 272 | 280 |

**Supplementary Datasheet S1**

**Parentage analysis**

Genotyping was carried out with the mother, her stored spermatangia and spawned eggs using four SSR markers (n = 4). All hatched paralarvae, every single spermatangium, i.e., six on left side, six on right side, stored by the female and her mantle tissue were subjected to DNA extraction followed by PCR and then fragment analysis. Microsatellite null alleles were eliminated from the analysis. The estimated size (bp) of each amplicon was shown. The data clearly show that in all microsatellite loci amplified from paralarval samples, one of two alleles was derived from spermatozoa stored in the spermatangia, and the other from the mother.
